# Maternal socio-economic status determines animal source food consumption of children aged 6–23 months in East African countries: a multilevel mixed-effects generalized linear model

**DOI:** 10.3389/fnut.2024.1336568

**Published:** 2024-10-17

**Authors:** Ibsa Mussa, Mulugeta Gamachu, Lemma Demissie Regassa, Abdi Birhanu, Fethia Mohammed, Alemayehu Deressa Weyessa, Addis Eyeberu, Adera Debela, Hamdi Fekredin Zakaria

**Affiliations:** ^1^School of Public Health, College of Health and Medical Sciences, Haramaya University, Harar, Ethiopia; ^2^School of Medicine, College of Health and Medical Sciences, Haramaya University, Harar, Ethiopia; ^3^Department of Psychiatry, School of Nursing and Midwifery, College of Health and Medical Sciences, Haramaya University, Harar, Ethiopia; ^4^School of Nursing and Midwifery, College of Health and Medical Sciences, Haramaya University, Harar, Ethiopia

**Keywords:** children aged 6–23 months, maternal socio-economic, multi-level analysis, animal source food consumption, East Africa

## Abstract

**Background:**

Nutrient deficiencies in Africa persist due to limited access to animal-source foods, causing a higher undernutrition prevalence, with 30.7% stunted children under five, which is higher than the global average of 22.0%. In African regions, there needs to be more information on access, consumption, and factors influencing children’s animal-source food consumption. Therefore, we comprehensively analyse data from the Demographic and Health Surveys (DHS) to determine factors associated with children’s (aged 6–23 months) consumption of animal-source foods (ASFs) in East African countries, which is crucial for policy and program development.

**Methods:**

We utilized cross-sectional pooled DHS data from nine East African countries reported from 2015 to 2021. The sample size consisted of 18,686 weighted children aged 6–23 months who were included. The DHS data were collected from women (15–49 years old) from households in each enumeration area of each country. We calculated the pooled proportion of consumption of foods of animal origin using multi-level logistic regression analysis to determine factors of ASFs, and the strength of the association was measured by an adjusted odds ratio (AOR) with a 95% CI and a *p*-value < 0.05, which was declared as significant.

**Result:**

The study found that 51.07% (95% CI: 50.26–51.88%) of infants and young children in East Africa consume ASFs, with variation across countries, of which 28.26% (95% CI: 26.31–30.29%) was the lowest in Burundi and 55.81% (95% CI: 53.39–58.21%) was the highest ASF consumption in Zimbabwe. The amount of ASF consumed grows with children’s age and varies greatly between countries. In addition, children in the wealthiest quintile and with the highest educational attainment consume more ASFs. However, those who lived in rural areas consumed fewer ASFs.

**Conclusion:**

The consumption of ASFs increased with the age of children, maternal education, and household wealth index. The government and non-government sectors should implement public health interventions targeting rural residents and poor households to increase access to and consumption of ASFs for children aged 6–23 months in East Africa.

## Introduction

Animal-source foods (ASFs) are crucial for nutrient-dense meals that include fish, meat, eggs, and dairy products. They also significantly lower the risk of undernutrition in vulnerable groups in resource-constrained environments, particularly for young children (1, 2). Many health-related issues, such as impaired cognitive functioning, poorer academic performance, morbidities, poverty, and over half of the under-five fatalities, have been linked to undernutrition (3). ASF is generally more nutrient- and energy-rich than nutrients obtained from plants (4). It has been discovered that older babies and young children who utilize ASFs increase dietary diversity, and there is strong evidence that a high level of dietary diversity significantly improves children’s nutritional outcomes (5). It has been recommended that newborns and early children start supplemental eating at six months old without quitting breast milk in order to enhance overall nutritional concerns (6, 7).

Two billion people worldwide suffer from micronutrient deficiencies, particularly those that come from eating meals made from animal products. Stunting affects 155 million children under the age of five globally, and millions more experience impaired cognitive development as a result of inadequate nutrition (2, 8). Although some parts of the world are making good progress in reducing malnutrition, in low and middle-income nations like African countries, nutrient deficiencies remain a public health concern due to the limited access and availability of ASFs for many infants and children, which is responsible for a higher prevalence of undernutrition, with about two out of every five children under the age of five (30.7%) being stunted, which is higher than the global average (9, 10). According to the study done by Ethiopian EDHS in 2016, only 8% of children aged 6–23 months consumed meat, fish, or poultry, and roughly 17 and 25% of children aged 6–23 months consumed eggs and dairy products, respectively. This means that only 14% of children meet the minimum dietary diversity requirements that consist of ASFs (11).

The consumption of minimally necessary food for infants and children is influenced by socioeconomic disparities such as place of residence, media exposure, and the educational status of families (12). Another study found that low income, reserving livestock for sales and ceremonies, living far from forested areas, high levels of small livestock morbidity and mortality resulting in a small or unstable flock or herd sizes, religion, child age, the number of household assets, ownership of land usable for agriculture, and the educational status of mothers were all barriers to accessing ASFs (9, 13, 14), villages being located near forested areas with wild animal populations, observing a large number of ceremonies of long duration, households with a greater number of small livestock, and where women can make autonomous decisions about livestock assets were factors associated with greater consumption of ASFs (13).

To address nutrition-related issues, particularly in young children under the age of five, the majority of African countries designed and implemented several multi-sectoral initiatives, including the National Nutrition Program and Food and Nutrition Policy strategies (15, 16). However, in resource-poor African countries, the availability and supply of animal-based food are insufficient, and as a result, the health and normal growth patterns of children are negatively impacted. There was little information about access, consumption, and related aspects of ASFs in African regions, particularly in East African countries. To design policies and programs for accomplishing the Sustainable Development Goal (SDG) of “zero hunger” by giving the issue of child undernutrition high attention, it may also be essential to examine the scope and influencing variables of ASF consumption in East African countries. Therefore, the current study sought to evaluate the extent and contributing factors of animal source food consumption among children in East African countries aged 6–23 months.

## Materials and methods

### Data source and study settings

The data was obtained from a secondary dataset that was created from the most current Demographic and Health Surveys (DHS) conducted in East African countries. The Demographic and Health Surveys are globally comparable household surveys that gather data from nationally representative samples of developing country households on demographic, socioeconomic, and health-related variables (17). The DHS initiative encompasses more than 90 low- and middle-income countries, which allows for comprehensive data collection. The program guaranteed uniformity by employing the same sampling technique, variable names, variable codes, manuals, and data-collecting instruments in each participating country (18). The study’s data came from the most recent DHS datasets, which were carried out in nine East African nations—Burundi, Ethiopia, Madagascar, Malawi, Rwanda, Tanzania, Uganda, Zambia, and Zimbabwe—between 2015 and 2021. These datasets were integrated to determine how children’s food consumption in East African nations is influenced by the socioeconomic status of their mothers.

### Sampling procedures and population

DHS employed a two-stage stratified cluster sampling approach, employing the Population and Housing Census (PHC) as the sample frame. In the first step, enumeration areas (EAs) were chosen using probability sampling with a proportional measure of the EAs’ size and independent selection in each sample stratum. In the second phase, a careful selection of households was made. An extensive sampling strategy was incorporated into the complete DHS report.

The DHS computation software was used to extract the data. A dataset on men, women, children, births, and households is included in every country’s survey. Kids’ record (KR) data, which includes details on households and types of food consumption, was used in this study. All of the selected households in the selected East African countries made up the study’s source population. Children who were excluded were those who were not expressly identified as possessing the outcome variable. Additionally, East African nations with insufficient sample sizes for the outcome variable were excluded. Consequently, a weighted sample of 18,686 participants who met the inclusion criteria were included in this study. Those that fit under this group include Burundi (1,964), Tanzania (3,132), Madagascar (1,861), Malawi (1,669), Ethiopia (2,923), Rwanda (1,203), Uganda (1,454), Zambia (2,846), and Zimbabwe (1,634).

### Measurement

The outcome variable of this study was the animal food source (AFS) of children aged 6–23 months. It was categorized into “0” (No ASF consumption) and “1” (ASF consumption). In the questionnaire, mothers or caregivers were asked what the child had eaten in the 24 h before the survey. The ASFs included in the questionnaire included eggs, fish, yoghurt, cheese, milk, and meat (including beef, poultry, hog, lamb, and any other meat not mentioned). Consumption of any quantity and/or kind of the aforementioned ASFs was regarded as a form of ASF consumption.

The independent variables included individual-level factors such as child age, child sex, respondent educational status, wealth index, access to media at least once a week, family size, place of delivery, antenatal visit and media exposure and community-level factors such as country and place of residence included in this study.

### Data management and analysis

Data from the nine East African nations were combined after the variables were extracted using the literature as a guide. Before any statistical analysis, the data were weighted using sampling weight, primary sampling unit, and strata to restore the representativeness of the survey and account for sampling design for generating standard errors and trustworthy estimates. Using STATA version 14, **StataCorp**. **2015**. Stata: Release 14. Statistical Software. Data extraction, recording, and analysis were carried out. Inferential and descriptive analyses were performed. Descriptive statistics, such as percent’s bar charts, pie charts, and frequency tables, were used to characterize the study, and tables, figures, and text were used to illustrate the findings.

The DHS data has a hierarchical structure for the determinant factors. The traditional logistic regression model’s assumptions of independent observations and equal variance are both violated by this. This suggests that when developing advanced models, it is necessary to account for the heterogeneity within clusters. This led to the fitting of a multilevel logistic regression model with both fixed and random effects to assess the factors associated with AFS consumption. Besides, to determine if there was clustering or not, the interclass correlation coefficient (ICC) and median odds ratio (MOR) were computed.

During a multilevel analysis, four models were fitted. Model II (adjusted for individual-level factors only), Model III (adjusted for community-level variables only), and Model IV (adjusted for both individual- and community-level variables concurrently) were among them. Among these was the null model, or model devoid of an independent variable. The optimal model for the data was then selected using the AIC and BIC (information criteria).

Variables used in multivariable analysis were chosen in bivariable analysis at a *p*-value of 0.2. To prevent the inflation of the effect size of independent variables, the variance inflation factor (VIF) was used to check for multi-collinearity. An adjusted odds ratio (AOR) with a 95% confidence interval was given in the multivariable analysis, and factors with a *p*-value of 0.05 or lower were regarded as significant determinants of the outcome variable. Finally, based on the Akaike information criterion (AIC) and Bayesian information criterion (BIC), the best-fitting model (model IV) was chosen among the fitted models.

### Ethical approval

We got the information from the webpage for the Demographic and Health Survey (DHS). Following registration, it can be accessed at: http://www.dhsprogram.com. The only aim of the information collection was to carry out a research study. We didn't reveal any specific families or individuals and kept all information confidential. DHS has received permission from the country and the National Research Ethics Review Committee (NRERC).

## Results

### Socio-demographic, economic, and maternal characteristics

A total of 18,686 children aged 6–23 months were included in this study. The mean age of the child, with a standard deviation, is 14.26 ± 5.13 months. Exactly half (50.0%) of the children’s mothers had a primary education. Nearly one-fourth of the households had the poorest wealth index status. The majority of the children, 14,453 (77.35%), were from rural areas. The largest number of children, 3,132 (16.76%), were from Tanzania, while the smallest number of children aged 6–23 months, 1,203 (6.44%), were from Rwanda (19, 20).

### Animal source food consumption magnitude in East Africa

**Figure 1:**
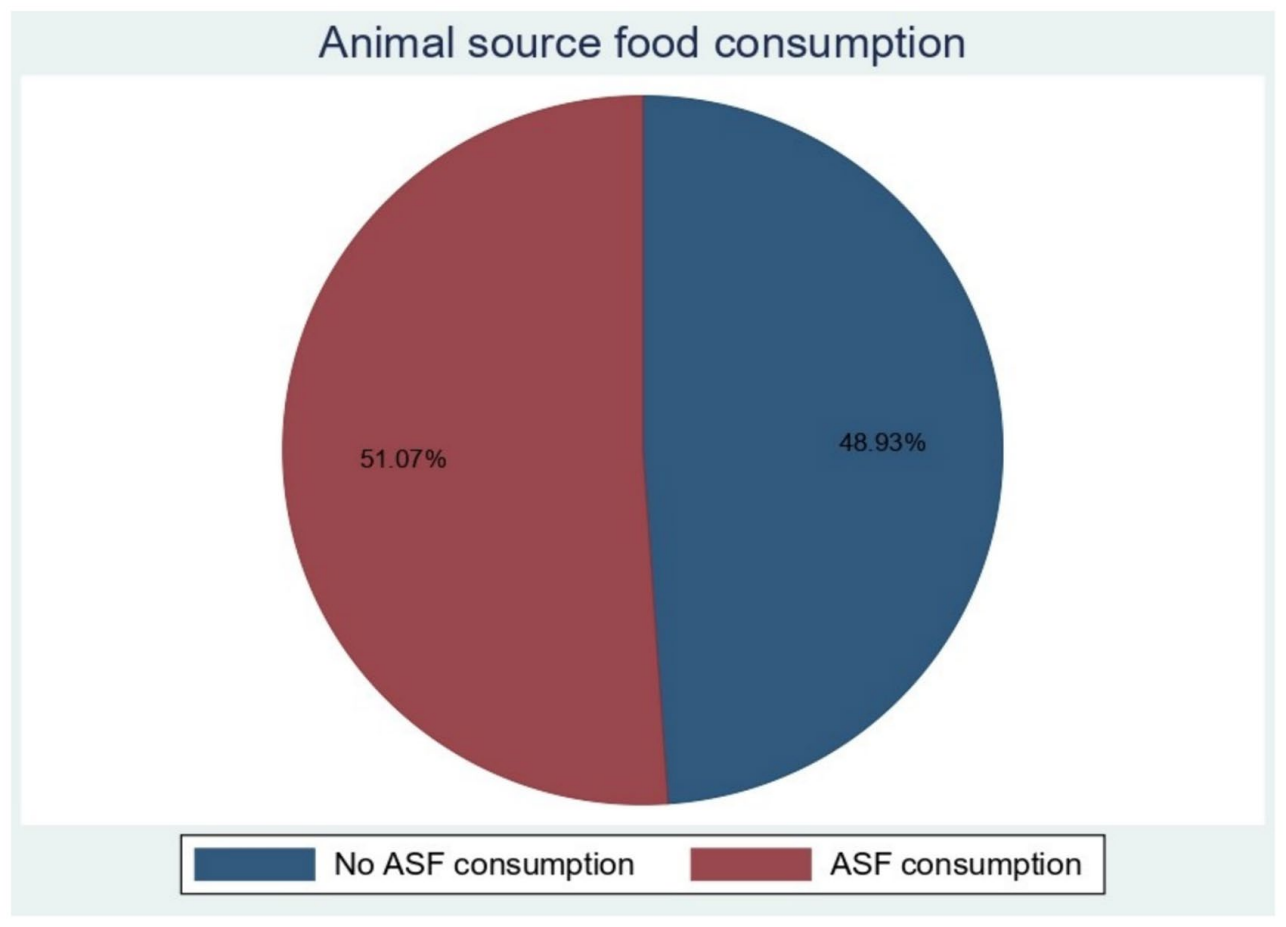
ASF consumption within 24-h consumption of animal food among children aged 6–23 months in East African countries from 2015 to 2021.

In this study, a total of 51.07% (95% CI: 50.26–51.88%) of infants and young children aged 6–23 months consumed ASF in East Africa, with variation between countries ranging from 28.26% in Burundi to 55.81% in Zimbabwe (Figure 1).

**Figure 2:**
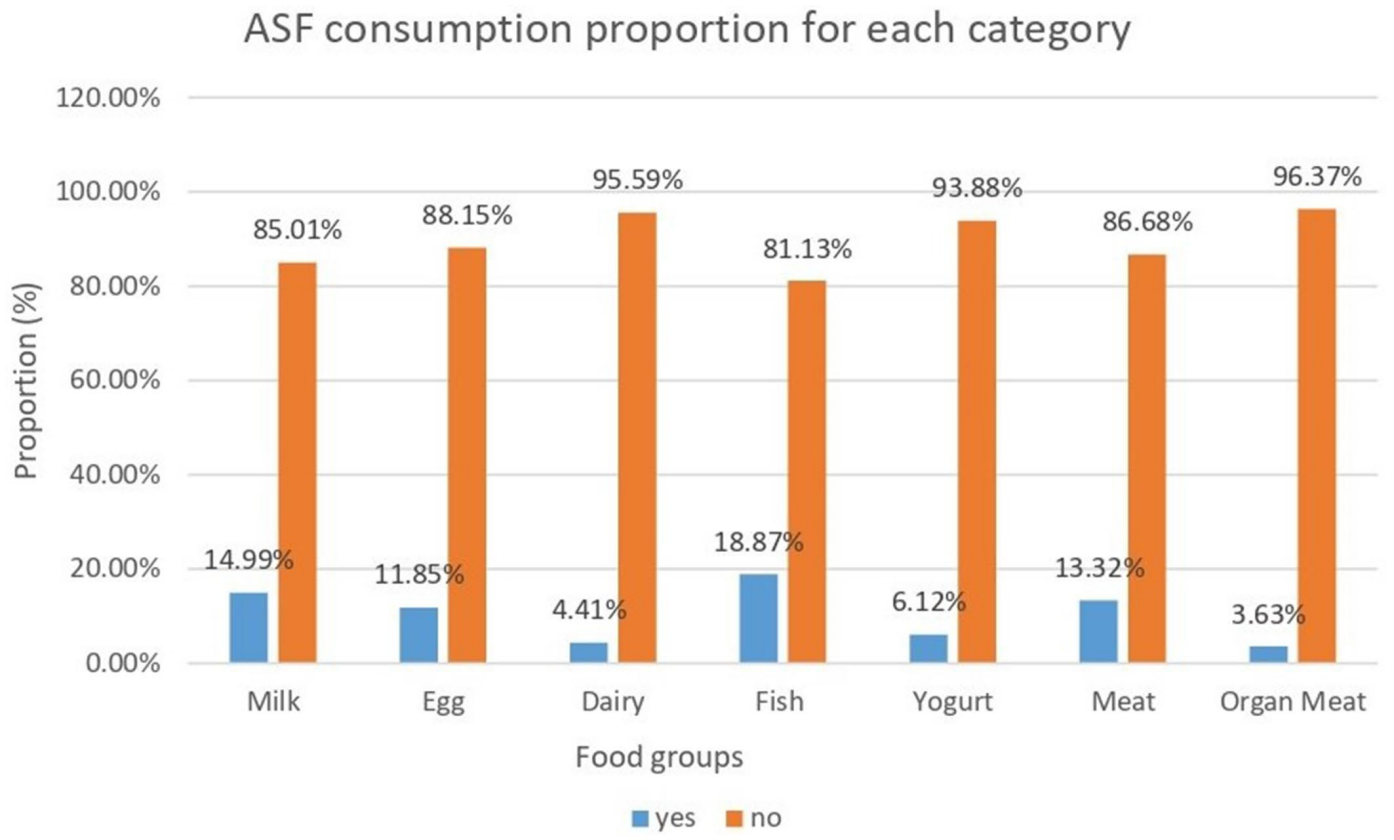
ASF consumption proportion for each category within 24-h consumption of animal food among children aged 6–23 months in East African countries from 2015 to 2021.

In the 24 h before the survey, 18.87, 11.85, and 14.99% of the children had fish, eggs, and milk, respectively. Meat consumption was reported by 13.32% of the study population. Very little dairy (4.41%), organ meat (3.63%), and yoghurt (6.12%) were consumed among the total number of children aged 6–23 months in East Africa (Figure 2).

### Determinants of animal food source consumption

Children aged 12–17 months [AOR 95% CI: 2.13 (1.92, 2.36)] and 18–23 months (AOR = 2.46; 95% CI: 2.21–2.74) consumed ASFs by more than two times as compared to younger children (6–8 months). Children from mothers who did attend education were 30% (AOR = 1.30; 95% CI: 1.17–1.44) more likely to consume ASF than children whose mothers did not have any education. The odds of consuming ASFs were also increased with the household wealth index, as children living in the richest households were about four times (AOR = 3.84; 95% CI: 3.27–4.51) more likely to consume ASFs compared to the poorest households, while the odds increased by two folds (AOR = 1.89; 95% CI: 1.67–2.14) for children from richer households. The odds of ASF consumption among those whose mothers had media exposure were higher by 24% (AOR = 1.24; 95% CI: 1.14–1.34) (see Table 1).

**Table 1:** Socio-demographic, economic, and maternal characteristics of 24-h consumption of animal food among children aged 6–23 months in the 11 East African countries from 2015 to 2021.

Variable	Weighted frequency	Percentage (%)
Child sex		
Male	9,411	50.36
Female	9,275	49.64
Child age in months		
6–8	3,224	17.25
9–11	3,133	16.77
12–17	6,557	35.09
18–23	5,772	30.89
Residence		
Urban	4,233	22.65
Rural	14,453	77.35
Respondent age in years		
15–24	6,714	35.93
25–34	8,293	44.38
35–49	3,679	19.69
Family size		
<=3	2,871	15.36
4–6	9,208	49.28
7 or above	6,607	35.36
Wealth index		
Poorest	4,804	25.71
Poorer	3,918	20.97
Middle	3,478	18.61
Richer	3,253	17.41
Richest	3,233	17.30
Respondent education		
No education	4,419	23.65
Any education	14,267	76.35
Husband education (*n* = 15,926)		
No education	3,319	20.84
Any education	12,607	79.16
Media exposure at least once a week		
No	11,848	63.41
Yes	6,838	36.59
Place of delivery		
Home	5,498	29.42
Health institution	13,188	70.58
4+ ANC visit		
No	8,302	45.92
Yes	9,778	54.08
Country		
Burundi	1,964	10.51
Ethiopia	2,923	15.64
Madagascar	1,861	9.96
Malawi	1,669	8.93
Rwanda	1,203	6.44
Tanzania	3,132	16.76
Uganda	1,454	7.78
Zambia	2,846	15.23
Zimbabwe	1,634	8.74

On the other hand, children who lived in rural areas were 20% less likely (AOR = 0.80; 95% CI: 0.77–0.95) to consume ASF compared to their counterparts. Furthermore, the consumption of ASFs varied among the countries included. Compared to Burundi, the consumption of ASF was significantly higher in Ethiopia (AOR = 3.57; 95% CI: 2.96–4.29) and Zambia (AOR = 4.04; 95% CI: 3.35–4.87) (Table 2).

**Table 2:** Multivariable multilevel mixed-effect logistic regression analysis of factors associated with 24-h consumption of animal food among children aged 6–23 months in East African countries from 2015 to 2021.

Variable	Model I (null model)	Model II AOR (95% CI)	Model-III AOR (95%CI)	Model-IV AOR (95%CI)
Child sex				
Male		1	1	1
Female		0.93 (0.87–0.99)		0.93 (0.87–1.05)
Respondent age in years				
15–24		1		1
25–34		1.01 (0.93–1.09)		1.05 (0.96–1.14)
35–49		0.89 (0.80–0.98)		0.95 (0.85–1.05)
Maternal education				
Any education		1.43 (1.29–1.57)		1.30 (1.17–1.44)*
No education		1		1
Wealth index				
Poorest		1		1
Poorer		1.06 (0.95–1.18)		1.09 (0.98–1.21)
Middle		1.25 (1.12–1.39)		1.28 (1.14–1.43)*
Richer		1.93 (1.71–2.18)		1.89 (1.67–2.14)*
Richest		4.28 (3.73–4.92)		3.84 (3.27–4.51)*
Family size				
<=3		1	–	1
4–6		0.94 (0.85–1.05)	–	0.93 (0.83–1.04)
Above 6		1.06 (0.94–1.19)	–	1.02 (0.91–1.14)
Media exposure				
No		1		1
Yes		1.24 (1.14–1.34)		1.24 (1.14–1.34)*
Child age in month				
6–8		1		1
9–11		1.67 (1.48–1.88)		1.68 (1.49–1.89)*
12–17		2.14 (1.93–2.38)		2.13 (1.92–2.36)*
18–23		2.47 (2.22–2.75)		2.46 (2.21–2.74)*
Place of delivery				
Home		1		1
Health institution		0.87 (0.79–0.96)		1.01 (0.92–1.11)
4+ ANC visit				
No		1		1
Yes		1.04 (0.97–1.12)		0.98 (0.91–1.05)
Residence				
Urban		–	1	1
Rural		–	0.35 (0.32–0.39)*	0.80 (0.70–0.91)*
Region				
Burundi			1	1
Ethiopia		–	2.94 (2.47–3.51)*	3.57 (2.96–4.29)*
Madagascar		–	3.14 (2.60–3.78)*	3.59 (2.95–4.38)*
Malawi		–	2.14 (1.77–2.58)*	2.09 (1.72–2.53)*
Rwanda		–	1.99 (1.62–2.45)*	1.93 (1.56–2.40)*
Tanzania		–	3.03 (2.54–3.61)*	3.15 (2.63–3.78)*
Uganda		–	3.24 (2.67–3.93)*	3.47 (2.83–4.25)*
Zambia		–	3.22 (2.70–3.86)*	4.04 (3.35–4.87)*
Zimbabwe		–	3.33 (2.73–4.07)*	3.49 (2.83–4.29)*

* =*p*, *p-value <0.05.

### Random effects result

The null model results, which are displayed in Table 3, show that there was statistically significant clustering of ASF, with a community variance (S.E.) of 1.1 (0.07). The null model’s ICC also demonstrated that differences between communities accounted for 25.09% of the total variance, and it ranged from 25.09% in the null model to 18.7% in the final model (model IV). ASF consumption was 2.71 times more likely to occur when respondents went from low-risk to high-risk neighborhoods, according to the MOR value of 2.71 (95% CI: 2.57–2.85), which is significant, and further suggests that there were significant differences in ASF consumption between clusters. The community variance (community variance = 0.75; SE = 0.05) was still significant but decreased in the full model (adjusted for individual and community characteristics).

**Table 3:** Community level variability and model fitness for assessment of the ASF of 24-h consumption among children aged 6–23 months in East Africa.

Parameter	Model I (null model)	Model II	Model III	Model IV
**Community variance (SE)**	1.10 (0.07)	0.91 (0.06)	0.78 (0.05)	0.75 (0.05)
ICC%	25.09%	21.76%	19.33%	18.7%
MOR	2.71	2.47	2.31	2.28
**Model comparison**				
AIC	25025.21	23069.53	24313.64	22724.49
BIC	25040.88	23209.98	24399.83	22935.16
Log-likelihood (LL)	−12,510.604	−11,516.765	−12,145.821	−11,335.247

Even after taking into consideration a few contextual risk variables, there was still a considerable difference in the amount of variance in ASF consumption that can be attributable to context—about 18.7% of it. Model IV, which had the lowest AIC and BIC, was the final model that fit the data the best in terms of model fitness (Table 3).

The interpretation of the fixed effects was based on the parsimonious model, which was determined to have lesser AIC and BIC. Compared to other models, Model-IV was adjusted for characteristics at the individual and community levels that have the smallest AIC and BIC, and this model fits the data. In the multivariable analysis, maternal education, household wealth index, media exposure, child age in a month, place of residence, and country of origin were significant determinants of animal-source foods.

## Discussion

In this study, the pooled magnitude of animal-source food consumption among 6-23-month-old children in East Africa was 51.07% (95% CI: 50.26–51.8%), ranging from 28.26% (95% CI: 26.31–30.29%) in Burundi to 55.81% (95% CI: 53.39–58.21%) in Zimbabwe. The current finding is comparable with a study done from DHS data from 49 countries (4); South, Central, and Southeast Asia (56.8%), West and Central Africa (52.0%), and Eastern and Southern Africa (49.3%). This finding is also in line with the study reported from four regions of Ethiopia (46.5%) (21) and the study conducted on 2016 DHS data in Ethiopia (51%) (9). However, this finding is lower than the findings reported from Latin America and the Caribbean (82.9%) and the Middle East and North Africa (75.5%). The discrepancy of this finding may be explained by the variation of socioeconomic, educational status, and geographic location of the countries, where countries with higher economic status reported higher consumption of animal-source foods, and those countries reported similar results to our finding were from low and middle-income regions. This suggests that children aged 6 to 23 months consume low-quality animal-sourced foods and should require appropriate regional and national-level interventions, particularly in eastern African countries, to reduce problems related to nutrient deficiency in children.

Factors such as the age of children, place of residence, educational status of mothers, wealth index of households, and media exposure of mothers were determinants of the consumption of ASFs among children aged 6 to 23 months in East Africa. Accordingly, children aged 18 to 23 months were 2.86 times more likely to consume animal-sourced food than children aged between 6 and 12 months. This finding was in line with the previous study done from 2016 EDHS in Ethiopia (9), northern Ethiopia (14), four regions of Ethiopia (21), and the study conducted in 49 world countries (4). This might be related to the fact that as the age of children increases, the demand for additional nutrients may increase, and the mother’s feeding skills may also improve as the child ages. Another possible explanation might be related to the attitude of families and other communities who feel infants and young children are not ready for meat and animal products until later ages. This implies that infants and young children were ignored, and parents should improve the feeding practices of children at an early age as animal-source foods are crucial for growth and development.

The odds of ASF consumption were higher by 30% among children whose mothers were educated compared to those who had no formal education. The finding was supported by previous studies conducted in Ethiopia and Cambodia (14, 22). This could be because maternal education may influence food choices and access to animal-source foods. Similarly, it can be linked with household income when educated mothers are in a better position to acquire better income, transportation, and market information, allowing them to purchase and provide affordable ASFs for their children (23). Moreover, maternal education indirectly improved the children’s dietary and nutritional outcomes through immediate effects on other characteristics such as household wealth and maternal employment.

The odds of animal-source food consumption among rural children aged 6 to 23 months were 15% less likely compared to their counterparts, which is supported by the studies conducted in 33 sub-Saharan African DHS (24) and India (25). The current study found that children living in rural areas were less likely to consume animal-source foods, even though natural animal-source foods are frequently available there. Similar to this, the 2020 Global Nutrition Report found that the location gap accounts for 4.9% of the global disparities in child nutrition (26). Additionally, it can be related to the educational difference and the lack of access to health information in rural areas, which might have an effect on women’s knowledge and experience about child feeding practices.

Children from parents who had media exposure were 24% more likely to consume ASFs compared to their counterparts. A similar finding was reported from a previous study done in Ethiopia (9). This might be due to parents determining what foods are available to their children and influencing their dietary habits. In addition, health information delivered by the media can indirectly influence children’s food choices by shaping parental attitudes and behaviors regarding their child’s feeding practice (9, 27).

Furthermore, the current study found that the odds of ASF consumption were 3.84 and 1.89 times higher among children from the richest and richer families, respectively, compared to those children who were from the poorest families. This finding is in line with the studies done in Ethiopia (14) and India (28). The possible justification for this finding could be that the poorest households struggle to afford diverse diets, including ASFs, due to limited financial resources. As a result, the poorest households experience food insecurity because they rely heavily on plant-based staples, resulting in limited dietary diversity and ASF intake (13).

### Strengths and limitations of the study

The main strength of this study is that it utilized a large sample size and that the sampling technique (especially the use of sampling weights) allowed every household to have an equal probability of inclusion. The larger sample size proved important in maintaining the internal validity of the study by helping provide precise descriptive and analytic findings. The other strength is that the data in this study were collected from East African countries, which increases the generalizability of the findings.

The cross-sectional design of this study means it does not show any cause-and-effect relationship between the outcome variable and the explanatory variables. Additionally, the lack of information on possible explanatory variables like the food insecurity status of the households may have changed the picture of the current associations between the variables. Therefore, incorporating a household food insecurity access score in future studies can be crucial in identifying other unseen associations. Another possible limitation is that this study did not provide information on the amount and frequency of ASF consumption. The use of 24-h dietary recalls may not perfectly tell about the usual diets (i.e., by creating within-person error), leading to an attenuation bias. Therefore, future similar studies should consider and try to address the points raised.

## Conclusion

The pooled magnitude of 24-h animal-source food consumption among 6–23 month-old children in East Africa was 51.07%, ranging from 28.26% in Burundi to 55.81% in Zimbabwe. Significant determinants of animal food source consumption included the wealth index, maternal education, household wealth index, media exposure, child age in a month, place of residence, and country of origin. It is crucial to concentrate on educating the mother of the children as well as strengthening nutritional counseling and nutrition education techniques using diverse media for increased access and consumption of ASFs to increase the intake of ASFs by children aged 6–23 months in East Africa. In addition, to improve and enhance the intake of ASF consumption among children aged 6–23 months, governmental and non-governmental organizations should design public health interventions on ASF consumption targeting rural residents and the poorest households.

## Data Availability

Publicly available datasets were analyzed in this study. This data can be found here: http://www.dhsprogram.com.
